# Hyperdiluting Calcium Hydroxylapatite With Platelet-Rich Plasma and Hyaluronidase for Improving Neck Laxity and Wrinkle Severity

**DOI:** 10.7759/cureus.63969

**Published:** 2024-07-06

**Authors:** Saami Khalifian, Alec D McCarthy, Steven G Yoelin

**Affiliations:** 1 Dermatology, SOM Aesthetics, San Diego, USA; 2 Medical Affairs, Merz Aesthetics, Raleigh, USA; 3 Department of Surgery: Transplant, University of Nebraska Medical Center, Omaha, USA; 4 Ophthalmology, Steve Yoelin MD Associates, Newport Beach, USA

**Keywords:** aesthetic medicine, cosmetic dermatology, regenerative aesthetics, hyaluronidase, platelet rich plasma, prp, caha, radiesse, calcium hydroxylapatite

## Abstract

The two cases discussed in this report investigate the efficacy and safety of a novel injectable therapy for treating neck wrinkles and skin laxity, utilizing a combination of hyperdiluted calcium hydroxylapatite (CaHA), platelet-rich plasma (PRP), and hyaluronidase. Two patients presenting with moderate neck wrinkles and laxity underwent treatment and were evaluated several months later. The combined therapy demonstrated improvements in skin texture and laxity following a single treatment. The rationale behind incorporating PRP and hyaluronidase was their potential to amplify the regenerative effects of CaHA. PRP contains growth factors that stimulate collagen production and tissue regeneration while hyaluronidase facilitates the breakdown of hyaluronic acid, promoting better diffusion and more even product dispersion. The findings from these cases provide emerging preliminary evidence supporting the safety and efficacy of this innovative combination therapy for addressing neck wrinkles and laxity. This is the first documented instance of skin priming CaHA with hyaluronidase and PRP. Future investigations are warranted to explore the application of this treatment for other anatomical regions and to delineate the role of each injected component.

## Introduction

Chronological and extrinsic aging, induced by factors such as ultraviolet (UV) or chemical damage, leads to the breakdown of the skin's extracellular matrix (ECM) [1,2]. This process is characterized by senescent fibroblasts, reduced production of ECM proteins, and diminished skin elasticity, resulting in increased skin laxity and wrinkles [3,4]. The neck, being particularly vulnerable to UV damage, commonly exhibits signs of aging such as neck wrinkles and skin laxity, which can be concerning for many individuals [5].

The treatment of cervical skin laxity and rhytids presents a significant challenge due to the limitations of current therapeutic modalities, despite the recent increases in medical research hoping to address this concern. Energy-based devices, such as radiofrequency and laser technologies, necessitate a delicate balance between delivering effective treatment without causing iatrogenic injury or scarring. Furthermore, traditional methods, such as hyaluronic acid (HA) dermal fillers and neurotoxins, have limitations (ie, simple volumetric displacement for HAs, suboptimal duration, and treatment area limited to platysma bands for neurotoxins) for treating neck laxity [6,7]. Their use in mitigating cervical rhytids also yields modest results, highlighting a glaring deficiency in our therapeutic armamentarium.

Given these challenges, there exists an unaddressed need for a safe, effective, and non-surgical solution for neck rejuvenation. This innovative approach aims to restore both the anatomical structure and the physiological function of the cervical region by augmenting soft tissue with regenerated collagen and elastin. Such a treatment should occur in a manner that ensures a minimal risk profile, with a focus on mitigating adverse events, while simultaneously delivering robust and satisfactory results. Few treatments are available to address this concern, though the use of regenerative aesthetic treatments such as calcium hydroxylapatite-carboxymethylcellulose (CaHA-CMC; Radiesse (+), Merz Aesthetics, Raleigh, NC) has emerged as an effective treatment option and has subsequently increased dramatically in public interest [8,9]. When diluted (1:1) or hyperdiluted (>1:2), CaHA-CMC is optimized for regeneration [10,11]. That is, the elastic modulus is dramatically reduced for optimal spread and a sufficient distance between microspheres allows for the mechanical interaction between CaHA microspheres and fibroblasts [10,12]. The CaHA microspheres contained in CaHA-CMC induce the regeneration of multiple components of the extracellular matrix (ECM), including collagens I and III, proteoglycans, and elastin by activating dermal fibroblasts [8,13-15]. Previous studies have quantified and correlated the increase in these proteins with improvements in the firmness (collagen), elasticity (elastin), and quality (proteoglycans) of the skin following hyperdilute CaHA-CMC treatment [8,15-17].

Recent studies have suggested that the combination of CaHA-CMC with skin priming agents, such as amino acids and/or exosomes, may enhance the regenerative outcome of the hyperdilute CaHA-CMC treatment [18,19]. One of the authors initially began mixing hyperdilute CaHA-CMC with hyaluronidase for better diffusion and more even spread in treating neck laxity. The other authors took this a step further by adding PRP into the hyperdilute CaHA-CMC-hyaluronidase mix. While treatment with hyperdilute CaHA-CMC is effective on its own, the addition of PRP and hyaluronidase may enhance the outcome by priming the skin with autologous growth factors. PRP contains a concentrated amount of growth factors that stimulate collagen production and tissue regeneration while hyaluronidase breaks down hyaluronic acid in the skin, allowing for better diffusion of the injected mixture and therefore more even product dispersion [20-23]. The combination of PRP and hyaluronidase as diluents for hyperdilute CaHA-CMC ensures the skin is primed with growth factors and that endogenous HA does not obstruct or constrict the even distribution of the product.

Reported here, for the first time to the best of our knowledge, is a treatment protocol combining hyperdiluted CaHA-CMC with PRP and hyaluronidase in the treatment of neck wrinkles in two Caucasian women, aged 56 and 72. The report provides the clinical methodology used and insight into the potential benefits of this novel approach and may inform future treatment options for patients with similar cosmetic concerns.

## Case presentation

Methodology

Prior to treatment, each patient was cleansed with chlorhexidine and dilute hypochlorous acid. Radiesse (+) was hyperdiluted 1:4 (1.5 ml CaHA-CMC and 6 ml of diluent). Of the 6 ml used to dilute the product, 1 ml was hyaluronidase (Hylenex recombinant 150 mg/mL, Halozyme Inc., San Diego, CA, USA) and 5 ml PRP (with at least 3x or greater concentration). To harvest PRP, the following steps were performed utilizing the ProGen Eclipse, a single-spin centrifuge system (ProGen Eclipse PRP, Dallas, Texas, USA). The patient’s blood was drawn into the ProGen 30 mL PRP tube, inverted seven times with anticoagulant mixing, and centrifuged within six minutes of blood draw at 1600 RCF for 10 min. Post-centrifugation, the platelet-poor plasma (PPP) was removed and the PRP was aspirated. The product was then ready for use as a diluent in the hyperdilute CaHA-CMC cocktail.

**Figure 1:**
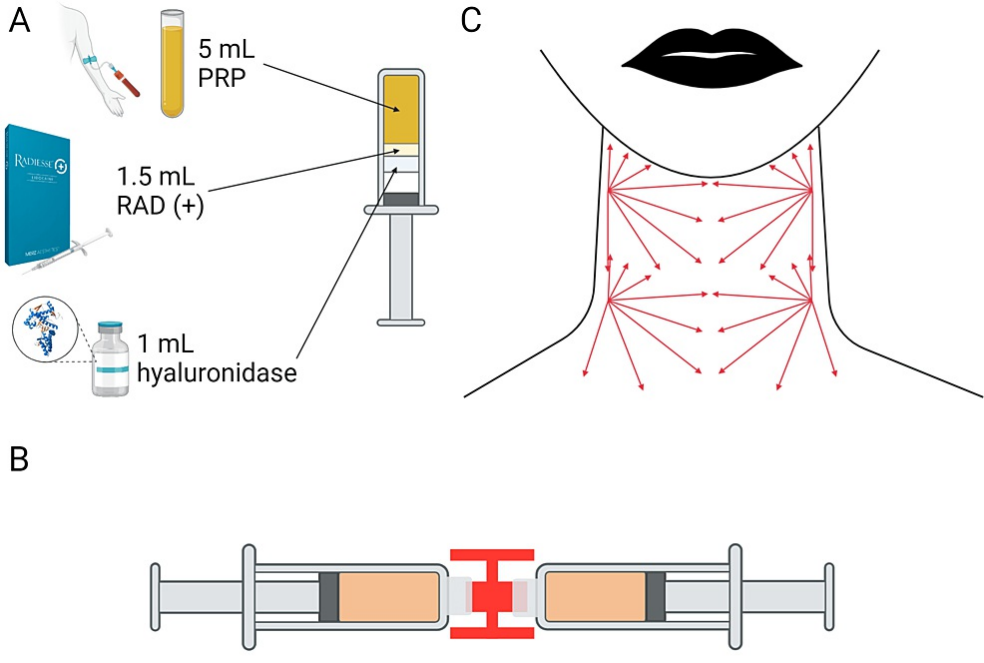
Preparation and protocol for neck treatment with CaHA-CMC/PRP (A) The components of a single syringe of treatment consisting of 5 ml autologous PRP, 1.5 ml CaHA-CMC, and 1 ml hyaluronidase; (B) The method for homogenizing the mixture (the resultant color of the solution is a light orange color); (C) The injection pattern used for both patients Figure credits: Original image created by the authors

After the autologous PRP was collected, one syringe (1.5 ml) of CaHA-CMC and 1 ml of hyaluronidase was transferred to a 10 ml syringe with a male-to-male Luer lock adaptor. An empty 10 ml syringe was attached to the Luer lock adaptor and the entire 7.5 ml solution (1.5 ml CaHA-CMC, 1.0 ml hyaluronidase, and 5 ml PRP) was passed between the two 10 ml syringes a minimum of 20 times (Figure 1). The resulting mixture was homogenous, as particle homogeneity is critical for the prevention of focal accumulations and nodules [24].

Local anesthetic was administered, ports were created with 23-gauge needles, and the product was placed using a 25 gauge, 1.5” cannula. The solution was injected in retrograde fanning patterns, with approximately 0.2 ml linear threads of the solution injected subdermal [9,25]. For patients with particularly bad laxity in the submentum, this area can also be treated with an additional series of fanning vectors. Following treatment, the neck was massaged, though patient self-massage is not required once the patient leaves the office, as the post-injection massage effectively distributes the CaHA microspheres [26]. The patients were photographed at follow-up, and written informed consent to publish the protocol, case data, and photographs was obtained.

To assess the efficacy of the treatment, nine reviewers (three clinical researchers, three plastic surgeons, and three dermatologists) were blinded and asked to grade each photo according to both the Merz Neck Laxity scale and the Lemperle Wrinkle Severity scale for both cases. The Lemperle Wrinkle Severity scale is a subjective scale with grades ranging from 0 to 5, with 0 indicating no wrinkles and 5 indicating very deep wrinkles. Similarly, the Merz Neck Laxity scale is a subjective scale ranging from 0 to 5, with 0 indicating no laxity and 5 indicating very severe laxity.

Cases

**Figure 2:**
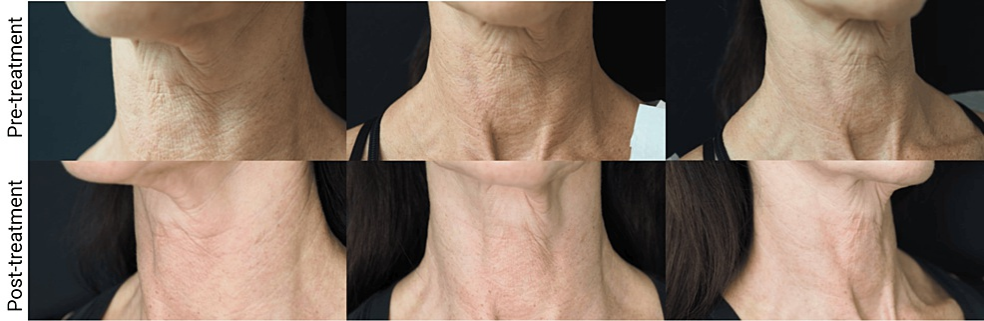
A 56-year-old patient 4 months after undergoing the described CaHA-CMC/PRP treatment CaHA-CMC: calcium hydroxylapatite-carboxymethylcellulose; PRP: platelet-rich plasma

In the first case, a 56-year-old Caucasian woman presented with concerns about neck wrinkling and skin laxity, attributed to significant sun damage throughout her life, especially in the neck and chest areas. No prior treatments for neck laxity were reported. The patient underwent a single treatment session with the novel hyperdilute CaHA-CMC preparation, receiving 7.5 ml of CaHA-CMC, PRP, and hyaluronidase injected subdermal using 4 fanning vectors. Post-treatment, the patient experienced no side effects. At the four-month follow-up, she had marked improvement in wrinkle severity and laxity, expressing high satisfaction with the outcome (Figure 2).

**Figure 3:**
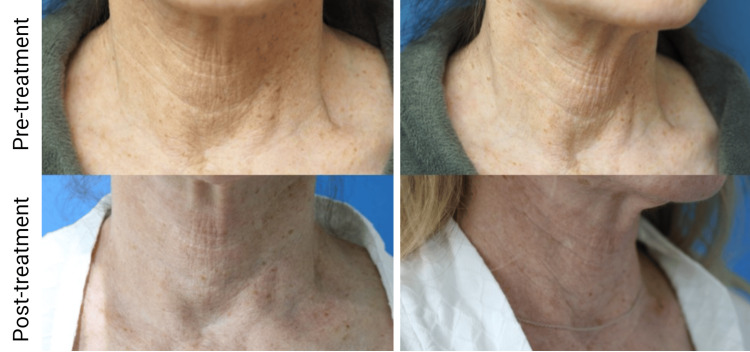
Figure 3. A 72-year-old patient 3 months after undergoing the described CaHA-CMC/PRP treatment CaHA-CMC: calcium hydroxylapatite-carboxymethylcellulose; PRP: platelet-rich plasma

In the second case, a 72-year-old Caucasian woman presented with similar concerns about neck wrinkling and skin laxity, also attributing it to significant sun damage. She had previously undergone underwhelming resurfacing and non-invasive neck tightening procedures. The patient underwent treatment with the novel hyperdilute CaHA-CMC preparation, receiving 7.5 ml of CaHA-CMC, PRP, and hyaluronidase injected subdermally using four fanning vectors. No adverse effects were observed post-treatment. At the three-month follow-up, she too had marked improvement in wrinkle severity and laxity and expressed high satisfaction with the single treatment (Figure 3).

In both cases, skin laxity and wrinkle severity improved. While the average neck laxity score pre-treatment was relatively low, both patients experienced improvements with average grades improving from a 2.0 to a 1.69. In contrast, the average pre-treatment wrinkle severity was high and improved following treatment from an average grade of 4.2 to 3.0 on the 5-point Lemperle Wrinkle Severity scale. Both patients expressed high degrees of satisfaction with the treatment and would recommend it, and neither patient reported any adverse effects associated with the treatment.

## Discussion

The combination therapy involving hyperdiluted CaHA-CMC with PRP and hyaluronidase presents a promising approach for treating mild to moderate neck wrinkles and laxity. This study offers preliminary evidence on the safety and efficacy of this treatment in two Caucasian women with significant neck skin wrinkles and moderate laxity. The treatment was well-tolerated, with no reported adverse events, and both patients expressed high satisfaction with the results following a single treatment.

The incorporation of PRP and hyaluronidase in the mixture may enhance the regenerative effects of CaHA-CMC by providing a concentrated dose of growth factors to the regenerating tissue. PRP's abundance of growth factors stimulates collagen production and tissue regeneration, leading to improved skin texture and reduced wrinkles [27]. Additionally, hyaluronidase's ability to break down hyaluronic acid in the skin facilitates better diffusion of the injected mixture, ensuring a more even distribution that may both prevent particle accumulation, but also more evenly distribute the injectable gel combination [28].

Several relevant factors should be considered during treatment planning and execution. First, it is imperative to consider the variability between PRP kits and centrifuges. Variability between brands and preparation techniques can result in significant variations in cell counts and PRP compositions [29]. This is important considering cell count is an important determinant in clinical outcomes [30]. To ensure reproducibility when using PRP, it is suggested to use standardized preparation methods and occasionally undergo hemocytometry to ensure an adequate cell count is consistently being achieved. Some biological parameters to consider are nicely summarized by Popescu et al. and include the volume of PRP obtained, relative PRP composition, recovery rate from whole blood, and activation status [31]. Next, the dilution of CaHA-CMC should be considered. While a 1:4 dilution was used in these reports, the dilution used should vary from patient to patient. Normal dilutions for CaHA-CMC neck treatments generally range from 1:2-1:4, but since PRP has inherent gel-like properties, dilutions may sway towards high dilutions (1:3-1:4) [32,33].

It is also important to acknowledge the limitations of this report, particularly the small sample size, which restricts the generalizability of the findings. The absence of a control group further hinders drawing definitive conclusions regarding the efficacy of the combination therapy compared to other treatments. To gain further insight, future studies with larger sample sizes and longer follow-up periods are required to investigate the long-term safety and efficacy of this novel injectable approach for treating neck wrinkles. Despite these limitations, the combination therapy of hyperdiluted CaHA-CMC with PRP and hyaluronidase offers a non-surgical solution for patients seeking effective and safe treatment for neck wrinkles.

## Conclusions

In conclusion, this case series investigated a novel combination therapy involving CaHA-CMC, PRP, and hyaluronidase for treating neck wrinkles in two Caucasian women. The results revealed that a single treatment session of this combination therapy was well-tolerated and improved skin texture while reducing neck wrinkles and laxity. The addition of PRP and hyaluronidase may amplify the regenerative effects of CaHA-CMC by supplying growth factors for tissue regeneration and enhancing product diffusion, respectively. Despite the limitations of a small sample size and the absence of a control group, this study offers preliminary evidence supporting the potential of this innovative approach. To further validate these findings and compare the efficacy with other treatments, future research with larger sample sizes and longer follow-up periods is warranted.

## References

[1] Fisher GJ, Kang S, Varani J, Bata-Csorgo Z, Wan Y, Datta S, Voorhees JJ (2002). Mechanisms of photoaging and chronological skin aging. Arch Dermatol.

[2] Varani J, Dame MK, Rittie L, Fligiel SE, Kang S, Fisher GJ, Voorhees JJ (2006). Decreased collagen production in chronologically aged skin: roles of age-dependent alteration in fibroblast function and defective mechanical stimulation. Am J Pathol.

[3] Reelfs O, Tyrrell RM, Pourzand C (2004). Ultraviolet a radiation-induced immediate iron release is a key modulator of the activation of NF-kappaB in human skin fibroblasts. J Invest Dermatol.

[4] Wlaschek M, Maity P, Makrantonaki E, Scharffetter-Kochanek K (2021). Connective tissue and fibroblast senescence in skin aging. J Invest Dermatol.

[5] Peterson JD, Goldman MP (2011). Rejuvenation of the aging chest: a review and our experience. Dermatol Surg.

[6] Ascher B, Talarico S, Cassuto D (2010). International consensus recommendations on the aesthetic usage of botulinum toxin type A (Speywood Unit)--Part II: Wrinkles on the middle and lower face, neck and chest. J Eur Acad Dermatol Venereol.

[7] Han TY, Lee JW, Lee JH (2011). Subdermal minimal surgery with hyaluronic acid as an effective treatment for neck wrinkles. Dermatol Surg.

[8] Yutskovskaya YA, Alexandrovna E (2017). Improved neocollagenesis and skin mechanical properties after injection of diluted calcium hydroxylapatite in the neck and décolletage: a pilot study. J Drugs Dermatol.

[9] Trindade de Almeida AR, Marques ER, Contin LA, Trindade de Almeida C, Muniz M (2023). Efficacy and tolerability of hyperdiluted calcium hydroxylapatite (Radiesse) for neck rejuvenation: clinical and ultrasonographic assessment. Clin Cosmet Investig Dermatol.

[10] Nowag B, Casabona G, Kippenberger S, Zöller N, Hengl T (2023). Calcium hydroxylapatite microspheres activate fibroblasts through direct contact to stimulate neocollagenesis. J Cosmet Dermatol.

[11] Casabona G, Pereira G (2017). Microfocused ultrasound with visualization and calcium hydroxylapatite for improving skin laxity and cellulite appearance. Plast Reconstr Surg Glob Open.

[12] Meland M, Groppi C, Lorenc ZP (2016). Rheological properties of calcium hydroxylapatite with integral lidocaine. J Drugs Dermatol.

[13] Yutskovskaya YA, Sergeeva AD, Kogan EA (2020). Combination of calcium hydroxylapatite diluted with normal saline and microfocused ultrasound with visualization for skin tightening. J Drugs Dermatol.

[14] Zerbinati N, Calligaro A (2018). Calcium hydroxylapatite treatment of human skin: evidence of collagen turnover through picrosirius red staining and circularly polarized microscopy. Clin Cosmet Investig Dermatol.

[15] González N, Goldberg DJ (2019). Evaluating the effects of injected calcium hydroxylapatite on changes in human skin elastin and proteoglycan formation. Dermatol Surg.

[16] Cogorno Wasylkowski V (2015). Body vectoring technique with Radiesse(®) for tightening of the abdomen, thighs, and brachial zone. Clin Cosmet Investig Dermatol.

[17] Kim J (2019). Multilayered injection of calcium hydroxylapatite filler on ischial soft tissue to rejuvenate the previous phase of chronic sitting pressure sore. Clin Cosmet Investig Dermatol.

[18] Theodorakopoulou E, McCarthy A, Perico V, Aguilera SB (2023). Optimizing skin regenerative response to calcium hydroxylapatite microspheres via biorevitalizing poly-micronutrient priming. J Drugs Dermatol.

[19] Choi MS, Park BC (2023). The efficacy and safety of the combination of photobiomodulation therapy and pulsed electromagnetic field therapy on androgenetic alopecia. J Cosmet Dermatol.

[20] Kim DH, Je YJ, Kim CD, Lee YH, Seo YJ, Lee JH, Lee Y (2011). Can platelet-rich plasma be used for skin rejuvenation? Evaluation of effects of platelet-rich plasma on human dermal fibroblast. Ann Dermatol.

[21] Redaelli A, Romano D, Marcianó A (2010). Face and neck revitalization with platelet-rich plasma (PRP): clinical outcome in a series of 23 consecutively treated patients. J Drugs Dermatol.

[22] Zhong SP, Campoccia D, Doherty PJ, Williams RL, Benedetti L, Williams DF (1994). Biodegradation of hyaluronic acid derivatives by hyaluronidase. Biomaterials.

[23] Chain E, Duthie ES (1940). Identity of hyaluronidase and spreading factor. Br J Exp Pathol.

[24] Lin JY, Hsu NJ, Lin CY (2022). Retaining even distribution of biostimulators microscopically and grossly: key to prevent non-inflammatory nodules formation. J Cosmet Dermatol.

[25] Guida S, Longhitano S, Spadafora M (2021). Hyperdiluted calcium hydroxylapatite for the treatment of skin laxity of the neck. Dermatol Ther.

[26] Casabona G, Alfertshofer M, Kaye K, Frank K, Moellhoff N, Davidovic K, Cotofana S (2023). Ex-vivo product distribution of injectable biostimulator substances. Aesthet Surg J.

[27] Banihashemi M, Nakhaeizadeh S (2014). An introduction to application of platelet rich plasma (PRP) in skin rejuvenation. Rev Clin Med.

[28] Shekhar C (2007). The matrix reloaded: Halozyme's recombinant enzyme helps injected drugs spread faster. Chem Biol.

[29] Magalon J, Bausset O, Serratrice N (2014). Characterization and comparison of 5 platelet-rich plasma preparations in a single-donor model. Arthroscopy.

[30] Tey RV, Haldankar P, Joshi VR, Raj R, Maradi R (2022). Variability in platelet-rich plasma preparations used in regenerative medicine: a comparative analysis. Stem Cells Int.

[31] Popescu MN, Iliescu MG, Beiu C, Popa LG, Mihai MM, Berteanu M, Ionescu AM (2021). Autologous platelet-rich plasma efficacy in the field of regenerative medicine: product and quality control. Biomed Res Int.

[32] Fabi SG, Alhaddad M, Boen M, Goldman M (2021). Prospective clinical trial evaluating the long-term safety and efficacy of calcium hydroxylapatite for chest rejuvenation. J Drugs Dermatol.

[33] Lorenc ZP, Black JM, Cheung JS (2022). Skin tightening with Hyperdilute CaHA: dilution practices and practical guidance for clinical practice. Aesthet Surg J.

